# International Multicenter Study on Drug Consumption in Nursing Students

**DOI:** 10.3390/ijerph18189526

**Published:** 2021-09-09

**Authors:** José Antonio Ponce-Blandón, José Manuel Martínez-Montilla, Manuel Pabón-Carrasco, Raúl Martos-García, Aurora Castro-Méndez, Rocío Romero-Castillo

**Affiliations:** 1 Spanish Red Cross Nursing School, University of Seville, Avda. de la Cruz Roja, nº 1 Dpdo., 41009 Seville, Spain; japonce@cruzroja.es (J.A.P.-B.); mpabon@cruzroja.es (M.P.-C.); rmartos@cruzroja.es (R.M.-G.); rocio.romero@cruzroja.es (R.R.-C.); 2 Department of Podiatry, School of Nursing, Physiotherapy and Podiatry, University of Seville, 41009 Seville, Spain; auroracastro@us.es

**Keywords:** drug users, substance abuse, students, nursing, universities, adolescent

## Abstract

Background: The prevalence of illicit drug use among young people is high, with many being highly vulnerable to substance abuse. The nursing profession is not immune to the impacts of substance misuse. Knowing the current levels of consumption of illicit drugs in nursing students will allow for the introduction of preventive actions. Methods: Multi-center, descriptive, and cross-sectional study involving nursing schools from four different countries (Spain, Belgium, France, and Brazil). A total of seven centers participated. An adapted version of the Global School-based Student Health Survey (GSHS) was used as a tool, which selected only the module on illicit drugs. Standard logistic regression analysis was performed. Results: A total of 496 nursing students participated in the study. Illicit drug shows positive representation among nursing students. A significant difference was observed between the gender and the age of first drug use, illicit drug consumption, cannabis use, and cocaine use ever in life, with higher use of illicit drugs by male, although at later ages than girls. The bivariate analysis, gender, problems as result of drugs, and nationality were significantly associated with the consumption of illicit drugs, cannabis, cocaine, and ecstasy ever in life. Conclusions: High rates of illicit drug use were found among nursing students, as well as factors that can influence consumption such as nationality or gender. These results can serve as a basis for the development of educational and policy interventions within nursing schools that are based on evidence, with significant implications for nurse educators, academic administrators, and practice.

## 1. Introduction

The consumption of illicit drugs represents an important public health problem worldwide [1], contributing to morbidity and mortality [2]. Nowadays, Europe has one of the highest percentages of students who reported lifetime use of any illicit drug, with 29% in the Czech Republic, followed by 28% in Italy. Besides, the most widely used illicit drug at least once in their life in Europe was cannabis (16%), followed by other illicit substances (5.0%), such as ecstasy, amphetamines, cocaine, and LSD, among others [3]. According to the survey on alcohol and other drugs in Spain (EDADES), cannabis is by far the most widespread in Spain, obtaining in 2019 the highest value in its history (37.5%), followed by cocaine (11.2%). In the population aged 15–34, the prevalence of illegal substance uses at least once in their life, in Spain, were cannabis (45%), cocaine (10.5%), ecstasy (5.4%), and amphetamines (4.2%) [4]. The use of these substances is associated with multiple consequences, including traffic accidents, violence, infectious diseases, mental disorder, impaired psychosocial development, and suicide, between others, particularly in the young population [5,6,7].

Different studies have shown several factors that have a special influence on illicit drug consumption use in young people [7,8,9]. Among the likely contributors to the elevation of drug use during adolescence and in young people are maturational changes [10]. In this line, in a study conducted in Spanish adolescents, the role of self-esteem and self-efficacy was highlighted, as was the importance of increasing the risk perception. Besides, the authors highlighted the social influence of the family and the people around them on binge drinking, highlighting the importance of increasing parents’ awareness factors, communication skills, adequate family influence, parental supervision, and control [11]. In addition, some authors also proposed that family environment and family support are key elements when starting to use drugs [12,13,14]. Moreover, there are studies that consider people from dysfunctional families to be at higher risk of consumption [8,15,16].

On the other hand, other authors highlight the importance of peer influences [11,17]. A weak relationship has been observed between gender and consumption with a higher prevalence of drug use among men in particular, with the exception of sedative hypnotics, where use is higher among women [7,8,18]. Furthermore, previous studies have shown that marital status may be related to substance use, although with mixed results [19,20]. Only some of the studies performed to date have taken the age of onset of consumption into account, despite the importance of this variable. Some studies suggest that age of onset is an important determinant of the magnitude of the consequences of drug use, in that younger individuals suffer greater health consequences [8,21].

In recent decades, health agencies and university authorities have expressed concerns over increasing alcohol consumption and other drugs of abuse such as cannabis and amphetamines among university students [22,23,24]. These students are at risk of substance abuse behaviors due to changes in lifestyle, reduced parental support, and stressful situations [25]. In this line, nursing students are also at risk for problems related to substance use [2,26].

The nursing profession is not immune to the impacts of substance misuse; in fact, rates of substance misuse among nurses are thought to mirror that of the general population [27]. Alcohol and other substance use by nurses potentially place patients, the public, and nurses themselves at risk for serious injury or death [26]. It is critical that rates of substance use in this population are elucidated and that risk factors are understood [2]. In addition, education studies offered by universities in health sciences should provide knowledge about the harmful health and social consequences of the use and abuse of drugs [28]. The knowledge of the toxic and social effects of drugs abuse is well known by university students; however, the ability to self-care for proper and good health may vary among people [29]. Thus, the high prevalence of illicit drug use among young people [3,22,23,24], as well as the fatal consequence of this use in health professionals, such as nurses who are in constant contact with patients [2,26], highlights the importance of preventing it in this target group.

Therefore, it is important to assess students’ consumption at the beginning of university period. While some studies evaluating illegal drug use in university populations have evaluated the prevalence of specific substances, such as cannabis or psycho stimulants, it would be advantageous to consider consumption more generally [8] in this target group [2,29]. The objective of this study was to understand the pattern of illicit drug use of nursing students from various world universities.

## 2. Materials and Methods

### 2.1. Study Design and Sample

This is a multi-center and cross-sectional study involving nursing schools from four different countries (Spain, France, Belgium, and Brazil). A total of seven centers participated: Red Cross Nursing Centre (Seville, Spain), Faculty of Nursing, Physiotherapy and Podiatry of University of Seville (Spain), Red Cross Nursing Centre, Toulouse (Toulouse, France), Haute Ecole Galilee (Brussels, Belgium), Faculty Anhanguera (São Paulo, Brazil), University Estadual Paulista Julio de Mesquita Filho (UNESP) (São Paulo, Brazil), and UNIFIPA, University Centre Padre Albino (São Paulo, Brazil).

A non-probabilistic sample was used along with an inclusion criterion age of less than 22 years old and the requirement that they were enrolled in the first or last year of nursing studies. As an exclusion criterion, to avoid confounding variables, repeating students of course were excluded. The sample size was calculated for a power of 0.95, with an alpha error of 0.05, and with medium size effect (G*Power, version 3.1.9.4, Franz Faul, Kiel University, Kiel, Germany) [30]. A total sample of 111 participants was estimated to be necessary.

### 2.2. Variables and Instruments

A Global School-based Student Health Survey (GSHS) was used as a tool [31]. This is a World Health Organization tool developed by the Centre for Disease Control in the USA. Drug consumption module was selected for this study. The following variables to know the illicit drug consumption was used: consumption of cannabis, cocaine, ecstasy, and amphetamines ever in life; and consumption of cannabis, cocaine, ecstasy, and amphetamines in the last 30 days. It consisted of eight questions answered on a 5-point Likert scale (0 = 0 times, 1 = 1–2 times, 2 = 3–9 times, 3 = 10–19 times, 4 = 20 or more times). Besides, the difficulty to obtain the different drugs was explored. It consisted of four questions answered on a 6-point Likert scale (0 = Impossible, 1 = Very difficult, 2 = Fairly difficult, 3 = Fairly easy, 4 = Very easy, 5= I do not know). It is a simple and self-applied survey to obtain data on the health behavior of the youths on the risk factors related to the main causes of mortality among children and adults worldwide. The survey was completed individually by each student. The data were completed on paper; subsequently, a researcher included the responses in a database. The GSHS is a collaborative surveillance project designed to help countries measure and assess the behavioral risk factors and protective factors in 10 key areas among young people. As previously mentioned, the drug use module was selected in this study. The survey was available in the four languages required according to the subjects of this study, English, Spanish, French and Portuguese.

In addition, social demographic variables were assessed at questionnaire, which consisted of gender (0 = male and 1 = female), age (in years), nationality (0 = Spain, 2 = Belgium, 3 = France, 4 = Brazil, and 5 = Others); center (0 = Faculty of Nursing of the University of Seville, 1 = Red Cross Nursing University, Seville, 2 = Haute Écolle Galilée, 3 = IRFSS French Red Cross, and 4 = University of Sao Paulo); course (0 = first year and 1 = last year); civil status (0 = single and 1 = married/common-law partner); age of first drug use (0 = I have never used drugs, 1 = 7 years old or younger, 2 = 8 or 9 years old, 3 = 10 or 11 years old, 4 = 12 or 13 years old, 5 = 14 or 15 years old, 6 = 16 or 17 years old, and 7 = 18 years old or older); problems with family, friends, school, or fights as a result of drugs (0 = 0 times, 1 = 1 or 2 times, 2 = 3 to 9 times, 3 = 10 to 19 times, and 4 = 20 or more times); and knowledge of the problem of drug use (0 = yes, 1 = no, and 2 = I do not know). No information was collected regarding comorbidity.

### 2.3. Procedures and Ethical Issues

The study observed the ethical considerations for research with human beings set forth in the Declaration of Helsinki and in the Law for the Protection of Personal Data. It was approved by the respective ethic committees of the participating centers. The first ethics committee that gave its approval was of the University of Seville, Spain. Subsequently, approval was obtained from all committees of the participating centers. All the students were previously informed and asked for their collaboration to fill out the questionnaire. This study did not require any intervention or experiment, only demographic data and information related to the consumption of alcohol were asked. All the participants gave their informed consent to participate in the study, and the voluntary nature of participation and the confidential handling of the data were highlighted.

### 2.4. Statistical Analyses

Descriptive analyses were performed to describe the characteristics of the participants, in which frequencies and percentages of the qualitative variables and measures of central tendency and dispersion for quantitative variables were obtained. Differences between the nationalities, as well as between academic year and gender, were assessed via a t-test for continuous variables and a chi-square test for categorical variables.

The complete sample (*n* = 496) was used to determine the variables associated with consumption ever in life of the different illicit drugs (cannabis, cocaine, ecstasy, and amphetamines), and the consumption in the last 30 days (cannabis, cocaine, ecstasy, and amphetamines). These variables were dichotomized (0 = non-consumption and 1 = consumption). In addition, the age variable of first drug use was reconverted to the illicit drug consumption in general, which was also dichotomized (0 = non-consumption and 1 = consumption). This variable only showed the consumption of general illicit drug in life. Standard logistic regression analysis was performed. The definitive model included variables with a *p* < 0.10. For the diagnosis, a Nagelkerke (R2CU) pseudo R2 value was calculated. The statistical tool used for data analysis was SPSS (IBM SPSS Statistics for Windows, Version 23.0. Armonk, NY, USA: IBM Corp.) version 23.

## 3. Results

A total of 496 students participated in the study; 403 (81.3%) participants were girls and the average age was 19.62 (standard deviation (SD) = 1.55). Most of the samples were Spanish students (*n* = 234; 47.2%), followed by French (*n* = 122; 24.6%) and Belgians (*n* = 70; 14.1%), and finally Brazilians (*n* = 53; 10.7%). There are a greater number of first-year students (*n* = 352; 71%), since not all of them finish their studies. The civil status shows that 469 participants were single (94.6%). According to the age of first illicit drug use, 179 (36.1%) participants consumed illicit drugs sometime in their life, 37 participants (7.5%) with 14 or 15 years old, 87 (17.5%) with 16 or 17 years old, and 48 (9.7%) with 18 years old or older. Most participants (*n* = 457; 92.1%) reported that they never had problems with their family, friends, schools, or fights as a result of drug use. Finally, 288 (58.1%) participants claimed that they have received knowledge of the problem of drug use by school.

### 3.1. Sample Characteristics

Characteristics of participants by gender are shown in Table 1. According to the academic year, nursing student girls were found in a higher proportion in the first year (*p* = 0.36). Regarding age of first drug use, males had higher illicit drug use, although later than girls (*p* = 0.010).

According to the drug use module, we found that boys were higher in illicit drug consumption (*p* = 0.001), such as cannabis use ever in life (*p* = 0.001), cannabis use in the last 30 days (*p* = 0.004), and cocaine use ever in life (*p* = 0.013).

On the other hand, characteristics of participants by academic year are shown in Table 2. According to the academic year, nursing students in their final year obtained a higher degree of knowledge than those in the first year (*p* = < 0.001). Besides, the problem with family, friends, school, or fight as a result of drugs use was marginally significant (*p* = 0.056), showing that the students in the last year had a high score.

### 3.2. Illicit Drug’s Use

According to the illicit drug use in nursing students, we found that the 179 (36.1%) had consumed illicit drugs throughout their whole life. Furthermore, 178 (35.9%) had consumed cannabis, 29 (5.8%) had consumed cocaine, 34 (6.9%) had consumed ecstasy, and 20 (4%) had consumed amphetamines. Besides, 90 (18.1%) had consumed cannabis, 10 (2%) had consumed cocaine, 11 (2.2%) had consumed ecstasy, and 7 (1.4%) had consumed amphetamines in the last 30 days.

In addition, we explore the difficulty of obtaining different illicit drugs, showing that obtaining illicit drugs is easy or very easy, such as cannabis (*n* = 298; 60.1%), cocaine (*n* = 171; 34.5%), ecstasy (*n* = 155; 31.3%), and amphetamines (*n* = 130; 26.2%). The illicit drugs module according to the Global School-Based Student Health Survey, as well as the difficulty of obtaining illicit drugs, are shown in Table 3 and Table 4, respectively.

Further, the illicit drug consumption between different nationality were also explored, where we found that France and Belgium reported high consumption regarding to the illicit drug ever in life (*p* ≤ 0.001), as well as cannabis, cocaine, and ecstasy consumption ever in life (*p* ≤ 0.001), and amphetamines consumption ever in life (*p* = 0.003). Besides, the consumption of cannabis in the last 30 days was also statically significant (*p* ≤ 0.001), with France and Belgium having the highest consumption. The illicit drugs consumption by nationality is shown in Table 5.

The logistic regression analyses are shown in Table 6. Nationality (*p* < 0.001) was significantly associated with the consumption of illicit drugs ever in life (no/yes), with Brazil being the country with the least consumption (*p* = 0.016), as well as gender (*p* = 0.001), with higher consumption by boys, and problems related to illicit drug use (*p* < 0.001) (never (*p* = 0.012) and ever (*p* < 0.001)). The model explained 25.5% of the variability associated with the use of illicit drugs ever in life (R2CU).

Regarding the consumption of cannabis ever in life (no/yes), nationality was significantly associated (*p* < 0.001), with Brazil being the country with the least consumption (*p* = 0.018), as well as gender (*p* = 0.001), with higher consumption by boys, and problems related to the use of illicit drugs (*p* < 0.001) (never (*p* = 0.013) and ever (*p* < 0.001)). The model explained that 25.1% of the variability was associated with ever-in-life cannabis use (R2CU). In addition, according to the consumption of cocaine ever in life (no/yes), age was statistically associated (*p* = 0.014), as well as gender (*p* = 0.012), with less consumption by girls (*p* = 0.043), as well as problems related to the use of illicit drugs (*p* < 0.001) (ever (*p* = 0.013)). Nationality emerged as a marginally variable which was statistically significant (*p* = 0.072). The model explained 35.3% of the variability associated with ever-in-life cocaine use (R2CU). Similarly, the consumption of ecstasy ever in life (no/yes), nationality (*p* = 0.002), as well as problems relating to the consumption of ecstasy (*p* < 0.001) (ever (*p* = 0.017)), were significantly associated. The model explained that 21.5% of the variability was associated with ever-in-life ecstasy use (R2CU). Finally, amphetamine consumption (no/yes) was significantly associated only with age (*p* < 0.001). The model explained 12.6% of the variability associated with ever-in-life amphetamine use (R2CU).

On the other hand, the logistic regression analysis was developed for the consumption in the last 30 days variables (cannabis, cocaine, ecstasy, and amphetamines). Only the variable of cannabis use in the last 30 days (no/yes) was statistically significant. For this analysis, the center (*p* < 0.001) (University of France and Belgium emerged as higher consumption of cannabis in the last 30 days), gender (*p* = 0.001) (with a higher consumption by boys), as well as problems related to illicit drug use (*p* < 0.001) (ever (*p* < 0.001)) were considered statistically significant. The model explained 26.3% of the variability associated with the use of illicit drugs ever in life (R2CU).

## 4. Discussion

In this paper, the pattern of illicit drug use of nursing students from various world universities was tested. For young people entering university for the first time, this stage is a period of maturation and change in health-related habits and lifestyles, including illicit drug use [8]. The nursing students are not immune to the impacts of substance misuse [27]; therefore, it is important to assess student consumption at the beginning of this period [8].

Regarding illicit drug patterns, this study is in line with other works carried out in Spain and Europe, where illicit drug shows positive representation among adolescents and young people [3,4,9]. In this sense, we found that 36.1% had consumed illicit drugs ever in life. Furthermore, 35.9% had consumed cannabis, 5.8% had consumed cocaine, 6.9% had consumed ecstasy, and 4% had consumed amphetamines ever in life. Besides, 18.1% had consumed cannabis, 2% had consumed cocaine, 2.2% had consumed ecstasy, and 1.4% had consumed amphetamines in the last 30 days. Currently, the effects of illicit drugs, both in the short and long term, are known [10,11]. In our study, most of participants reported that they have never had problems with their family, friends, schools, or fights as a result of drugs use, emerging as a statistically significant variable in the bivariate analysis. However, some studies highlight that the use of cannabis and cocaine was related to problems in school, unjustified absences, and repetition of a grade, as well as polyconsumption [32].

Characteristics of illicit drug patterns by gender are also in line with previous studies, whereby illicit drug use was associated with males in a higher proportion [7,8]. We found that boys had a higher use of illicit drugs, although at later ages than girls, such as cannabis use ever in life, cannabis use in the last 30 days, and cocaine use ever in life, which emerged as a significant association. This is a relevant finding considering that most of the students in the study sample were women studying nursing, and that the opposite was expected, as in the previous study by Colomer-Perez et al. [29]. This suggests that female students possibly faced a perceived higher risk than males for all substances [32], or instead they may be more knowledgeable and practice healthy habits, in contrast to other studies, which suggest that male students had more heathy habits than girls [29], i.e., sedentary and overweight lifestyles which are more prevalent among female students compared to male students [33]. A qualitative study showed that gender was not a differentiating factor in attitudes towards drug use. Influencing factors were cultural differences between students [34].

According to the age of first illicit drug use, in our study, 7.5% of participants had consumed illicit drugs at 14–15 years old, 17.5% at 16–17 years old, and 9.7% at 18 years old or older, being in line with previous study in which the age of first illicit drug use were around 15 years old [32]. In addition, we explored the difficulty of obtaining different illicit drugs. More than half of the students claimed that obtaining cannabis was easy or very easy. Instead, only around 30% of students claimed that obtaining cocaine, ecstasy, and amphetamines was easy or very easy. Our data on student perceived availability showed higher percentages than previous studies in Europe, where cannabis was also the most widely available drug (32%), followed by ecstasy (14%), cocaine (13%), amphetamine (10%), and methamphetamine (8.5%) [3]. In addition, we did not find any different by gender; instead, according to a ESPAD study, boys were more likely to consider cannabis to be easily available than girls [3].

Most of the students were single, which is very common for those living at home with their parents at this age. Some studies showed that living with the family is a protective factor and working during university studies is detrimental to risky consumption compared to living with roommates or alone, which supports previous studies carried out with students [29,35]. Interventions to counteract these risk behaviors should also include strengthening prosocial participation and parental control. Likewise, we consider it important in future studies to take into account other variables not considered in our analyses such as place of residence (student residences or at home with parents), family composition, family consumption, self-perception of family functional status (APGAR), family support, and social class, among others. These variables could be related to drug use in the university student population (as protective or risk factors) since various studies highlight the family as a factor to take into account in the use of adolescents and young people [11,12,13,14].

On the other hand, the illicit drug consumption between different nationalities were also explored; we found that France and Belgium reported high consumption regarding to the illicit drug ever in life, as well as cannabis, cocaine, ecstasy, and amphetamines consumption ever in life. Besides, the consumption of cannabis in the last 30 days was also statically significant, with France and Belgium being the ones with the highest consumption. This was in line with the bivariate analysis, in which nationality were significantly associated with the consumption of illicit drugs, cannabis, and ecstasy ever in life. According to the ESPAD study (2020), France had more consumption in any drug (24%), following by Spain (23%) and Belgium (18%). Cannabis use was the same in Spain and France (23%), being lower in Belgium (17%). Despite this, Belgium presented higher consumption of ecstasy and amphetamines than Spain and France [3]. Due to the differences previously found between countries, this could allow a broader vision to develop more specific drug use prevention programs [8,36].

Finally, only 58% of nursing students claimed to have received knowledge of the problem of illicit drug use at university; this may be explained by the fact that there are a greater number of first-year students, and they have not yet acquired the knowledge. Despite this, previous studies showed that nursing students have little knowledge of different drugs, as well as the potential uses of medical marijuana, and the risks associated with it [37]. In this line, some authors claimed specific risk factors for drug use in nursing students, increased stress, and anxiety in the context of academic workload and lack of education on addiction or alcohol within schools of nursing curricula [2]. Furthermore, a study by Strobbe et al. [26] included risk factors for the use of illicit drugs in nursing students, the lack of education about the use of substances, inconsistent policies and procedures, and insufficient available interventions. This appears contrary to what is expected of these health professionals, since those who study health sciences should be the best candidates to acquire self-care skills and behave in a way that limits the adverse consequences for the health of inappropriate lifestyles [29]. Substance misuse in nursing students may have more widespread negative sequelae, when compared to other students, since the impacts of substance misuse in nurses and nursing students have the potential to not only affect the individual but also the patients that they may care for [2,26].

Despite this, the risk factors studied are not specific to nursing students. There is a profound paucity in the literature regarding to the prevalence of substance use among nursing students, as well as its risk factors [2]. Therefore, to reduce the risk of nursing students and later newly graduated nurses in the use of drugs, larger and long-term studies should be carried out to understand the factors that influence the use of illicit drugs in nursing students, including the previously mentioned variables, so as to facilitate the implementation of evidence-based interventions within nursing schools [38].

### Limitations

In terms of the limitations of this study, the cross-sectional nature is noted, which could cause biases inherent to this type of model, since they do not establish the temporality between the exposure and the result. Another limitation is the high percentage of female subjects, due to the fact that nursing studies are coursed by a predominantly female population. In addition, data collection through surveys raises different biases that have tried to alleviate using trained interviewers in an environment of confidentiality and anonymity. The possibility of information bias must also be considered, as students may tend to respond positively. This study only focuses on nursing students, so the conclusions cannot be extended to health sciences students. It is a proposal for future study to include students from other disciplines such as medicine and physiotherapy, especially in professions related to health promotion.

Finally, it should be noted that we were unable to use a probability sampling approach, which may affect our inferences about the student population of this sample and the corresponding results, which, together with the responses that were not answered by the students and those who did not wish to participate, constitute selection bias. We tried to compensate for this bias with the large amount of sample collected in different countries, which is one of the strengths of our study.

## 5. Conclusions

The results of this study have significant implications for nurse educators and academic administrators, with clinical importance. High rates of illicit drug use were found among nursing students, as well as factors that can influence consumption such as nationality or gender. These evidence-based results can guide and support the development of educational and policy interventions within nursing schools which are able to screen students for the presence of substance use problems with strong referral networks established to ensure access to treatment.

## Figures and Tables

**Table 1:** Characteristics of participants. Differences by gender.

Variable (Number of Missing Values)		Total (*n* = 496)	Male (*n* = 89)	Female (*n* = 403)	Test Statistic	*p* Value
**Age (15–19-year-olds) (0), mean (SD)**		19.62 (1.55)	19.56 (1.790)	19.63 (1.503)	*t*_116.906_ = −0.348	0.729
**Nationality (0)**					χ^2^_4_ = 13.297	0.102
	Spain	234 (47.2)	53 (59.6)	179 (44.4)		
	Belgium	70 (14.1)	12 (13.5)	58 (14.4)		
	France	122 (24.6)	18 (20.2)	103 (25.6)		
	Brazil	53 (10.7)	2 (2.2)	50 (12.4)		
	Others	17 (3.4)	4 (4.5)	13 (3.2)		
**Centre (0)**					χ^2^_4_ = 14.185	0.077
	Faculty of Nursing of the University of Seville	155 (31.3)	36 (40.4)	118 (29.3)		
	Red Cross Nursing University, Seville	77 (15.5)	17 (19.1)	59 (14.6)		
	Haute Écolle Galilée	171 (34.5)	28 (31.5)	143 (35.5)		
	IRFSS French Red Cross	39 (7.9)	5 (5.6)	33 (8.2)		
	University of Sao Paulo	54 (10.9)	3 (3.4)	50 (12.4)		
**Academic year (8)**					χ^2^_1_ = 10.268	0.036
	First year	352 (71)	72 (80.9)	279 (69.2)		
	Last year	136 (27.4)	17 (19.1)	116 (28.8)		
**Civil Status (9)**					χ^2^_1_ = 2.312	0.679
	Single	469 (94.6)	86 (96.6)	379 (94)		
	Married/Common-law partner	18 (3.6)	3 (3.4)	15 (3.7)		
**Age of first drug use (0)**					χ^2^_7_ = 23.251	0.010
	I have never used drugs	317 (63.9)	42 (47.2)	272 (67.5)		
	7 years old or younger	0 (0)	0 (0)	0 (0)		
	8 or 9 years old	3 (0.6)	2 (2.2)	1 (0.2)		
	10 or 11 years old	0 (0)	0 (0)	0 (0)		
	12 or 13 years old	4 (0.8)	0 (0)	4 (1)		
	14 or 15 years old	37 (7.5)	6 (6.7)	31 (7.7)		
	16 or 17 years old	87 (17.5)	27 (30.3)	59 (14.6)		
	18 years old or older	48 (9.7)	12 (13.5)	36 (8.9)		
**Problems with family, friends, school or fights as a result of drugs (15)**					χ^2^_4_ = 11.416	0.326
	0 times	457 (92.1)	79 (88.8)	374 (92.8)		
	1 or 2 times	15 (3)	2 (2.2)	13 (3.2)		
	3 to 9 times	3 (0.6)	1 (1.1)	2 (0.5)		
	10 to 19 times	4 (0.8)	3 (3.4)	1 (0.2)		
	20 or more times	2 (0.4)	1 (1.1)	1 (0.2)		
**Knowledge of the problem of drug use (19)**					χ^2^_2_ = 3.510	0.743
	Yes	288 (58.1)	52 (58.4)	233 (57.8)		
	No	155 (31.3)	31 (34.8)	123 (30.5)		
	I do not know	34 (6.9)	5 (5.6)	29 (7.2)		
**Drug Use Module (0)**						
	Illicit drug consumption	179 (36.1)	47 (52.8)	131 (32.5)	χ^2^_1_ = 13.243	0.001
	Cannabis/marijuana/hashish use ever in life	178 (35.9)	47 (52.8)	130 (32.3)	χ^2^_1_ = 13.589	0.001
	Cannabis/marijuana/hashish use in the last 30 days	90 (18.1)	27 (30.3)	62 (15.4)	χ^2^_1_ = 11.101	0.004
	Cocaine use ever in life	29 (5.8)	10 (11.2)	18 (4.5)	χ^2^_1_ = 8.756	0.013
	Cocaine use in the last 30 days	10 (2)	3 (3.4)	7 (1.7)	χ^2^_1_ = 1.068	0.586
	Ecstasy/MDMMDMA use ever in life	34 (6.9)	10 (11.2)	24 (6)	χ^2^_1_ = 3.481	0.175
	Ecstasy/MDMMDMA use in the last 30 days	11 (2.2)	4 (4.5)	7 (1.7)	χ^2^_1_ = 2.647	0.266
	Amphetamines/methamphetamine use ever in life	20 (4)	7 (7.9)	13 (3.2)	χ^2^_1_ = 4.224	0.121
	Amphetamines/methamphetamine use in the last 30 days	7 (1.4)	3 (3.4)	4 (1)	χ^2^_1_ = 3.021	0.221

**Table 2:** Characteristics of participants. Differences by academic year.

Variable (Number of Missing Values)		Total (*n* = 496)	First Year (*n* = 352)	Last Year (*n* = 136)	Test Statistic	*p* Value
**Age of first drug use (0)**					χ^2^_7_ = 4.350	0.930
	I have never used drugs	317 (63.9)	225 (63.9)	86 (63.2)		
	7 years old or younger	0 (0)	0 (0)	0 (0)		
	8 or 9 years old	3 (0.6)	3 (0.9)	0 (0)		
	10 or 11 years old	0 (0)	0 (0)	0 (0)		
	12 or 13 years old	4 (0.8)	2 (0.6)	2 (0.6)		
	14 or 15 years old	37 (7.5)	25 (7.1)	11 (8.1)		
	16 or 17 years old	87 (17.5)	63 (17.9)	24 (17.6)		
	18 years old or older	48 (9.7)	34 (9.7)	13 (9.6)		
**Problems with family, friends, school or fights as a result of drugs (15)**					χ^2^_4_ = 17.967	0.056
	0 times	457 (92.1)	324 (92)	126 (92.6)		
	1 or 2 times	15 (3)	9 (2.6)	6 (4.4)		
	3 to 9 times	3 (0.6)	3 (0.9)	0 (0)		
	10 to 19 times	4 (0.8)	2 (0.6)	1 (0.7)		
	20 or more times	2 (0.4)	2 (0.6)	0 (0)		
**Knowledge of the problem of drug use (19)**					χ^2^_2_ = 66.356	<0.001
	Yes	288 (58.1)	167 (47.4)	118 (86.8)		
	No	155 (31.3)	141 (40.1)	10 (7.4)		
	I do not know	34 (6.9)	29 (8.2)	4 (2.9)		
**Drug Use Module (0)**						
	Illicit drug consumption	179 (36.1)	127 (36.1)	50 (36.8)	χ^2^_1_ = 0.453	0.797
	Cannabis/marijuana/hashish use ever in life	178 (35.9)	126 (35.8)	50 (36.8)	χ^2^_1_ = 0.459	0.795
	Cannabis/marijuana/hashish use in the last 30 days	90 (18.1)	63 (17.9)	25 (18.4)	χ^2^_1_ = 0.273	0.873
	Cocaine use ever in life	29 (5.8)	19 (5.4)	9 (6.6)	χ^2^_1_ = 0.919	0.632
	Cocaine use in the last 30 days	10 (2)	7 (2)	3 (2.2)	χ^2^_1_ = 0.191	0.909
	Ecstasy/MDMMDMA use ever in life	34 (6.9)	21 (6)	11 (8.1)	χ^2^_1_ = 4.885	0.087
	Ecstasy/MDMMDMA use in the last 30 days	11 (2.2)	5 (1.4)	6 (4.4)	χ^2^_1_ = 4.232	0.121
	Amphetamines/methamphetamine use ever in life	20 (4)	13 (3.7)	7 (5.1)	χ^2^_1_ = 0.877	0.645
	Amphetamines/methamphetamine use in the last 30 days	7 (1.4)	3 (0.9)	4 (2.9)	χ^2^_1_ = 3.193	0.203

**Table 3:** Illicit drugs use module based on Global School-Based Student Health Survey.

Drug Use Module					
Variables	0 Times	1–2 Times	3–9 Times	10–19 Times	20 or More Times
(mX): Missing Values per Variable	*n* (%)	*n* (%)	*n* (%)	*n* (%)	*n* (%)
**During your life, how many times have you used cannabis/marijuana/hashish (0)**	318 (64.1)	42 (8.5)	49 (9.9)	20 (4)	67 (13.5)
**During the past 30 days, how many times have you used cannabis/marijuana/hashish (0)**	406 (81.9)	45 (9.1)	17 (3.4)	14 (2.8)	14 (2.8)
**During your life, how many times have you used cocaine (0)**	467 (94.2)	15 (3)	8 (1.6)	3 (0.6)	3 (0.6)
**During the past 30 days, how many times have you used cocaine (0)**	486 (98)	8 (1.6)	1 (0.2)	1 (0.2)	0 (0)
**During your life, how many times have you used ecstasy/MDMMDMA (0)**	462 (93.1)	14 (2.8)	9 (1.8)	7 (1.4)	4 (0.8)
**During the past 30 days, how many times have you used ecstasy/MDMMDMA (0)**	485 (97.8)	9 (1.8)	0 (0)	1 (0.2)	1 (0.2)
**During your life, how many times have you used amphetamines/methamphetamine (0)**	476 (96)	11 (2.2)	5 (1)	3 (0.6)	1 (0.2)
**During the past 30 days, how many times have you used amphetamines/methamphetamine (0)**	489 (98.6)	5 (1)	1 (0.2)	0 (0)	1 (0.2)

**Table 4:** Difficulty of obtaining drugs based on Global School-Based Student Health Survey.

Difficulty of Obtaining Drugs						
Variables	Impossible	Very Difficult	Fairly Difficult	Fairly Easy	Very Easy	I Do Not Know
(mX): Missing Values per Variable	*n* (%)	*n* (%)	*n* (%)	*n* (%)	*n* (%)	*n* (%)
**How difficult do you think it would be for you to get cannabis/marijuana/hashish (0)**	17 (3.4)	25 (5)	39 (7.9)	170 (34.3)	128 (25.8)	117 (23.6)
**How difficult do you think it would be for you to get cocaine (0)**	26 (5.2)	49 (9.9)	81 (16.3)	119 (24)	52 (10.5)	169 (34.1)
**How difficult do you think it would be for you to get ecstasy/MDMMDMA (0)**	33 (6.7)	52 (10.5)	71 (14.3)	108 (21.8)	47 (9.5)	185 (37.3)
**How difficult do you think it would be for you to get amphetamines/methamphetamine (0)**	26 (5.2)	62 (12.5)	69 (13.9)	89 (17.9)	41 (8.3)	209 (42.1)

**Table 5:** Illicit drug use by nationality.

Drugs Consumption by Nationality							
Variables	Spain (*n* = 234)	Belgium (*n* = 70)	France (*n* = 122)	Brazil (*n* = 53)	Others (*n* = 17)	Test Statistic	*p* Value
(mX): Missing Values per Variable	*n* (%)	*n* (%)	*n* (%)	*n* (%)	*n* (%)		
**Drug consumption (0)**	64 (27.4)	31 (44.3)	72 (59)	8 (15.1)	4 (23.5)	χ^2^_1_ = 48.882	<0.001
**Cannabis/marijuana/hashish use ever in life (0)**	66 (28.2)	30 (42.9)	71 (58.2)	7 (13.2)	4 (23.5)	χ^2^_1_ = 46.848	<0.001
**Cannabis/marijuana/hashish use in the last 30 days (0)**	24 (10.3)	21 (30)	38 (31.1)	3 (5.7)	4 (23.5)	χ^2^_1_ = 36.209	<0.001
**Cocaine use ever in life (0)**	1 (0.4)	8 (11.4)	17 (13.9)	2 (3.8)	1 (5.9)	χ^2^_1_ = 31.356	<0.001
**Cocaine use in the last 30 days (0)**	3 (1.3)	1 (1.4)	5 (4.1)	0 (0)	1 (5.9)	χ^2^_1_ = 5.815	0.213
**Ecstasy/MDMMDMA use ever in life (0)**	7 (3)	4 (5.7)	20 (16.4)	2 (3.8)	1 (5.9)	χ^2^_1_ = 23.811	<0.001
**Ecstasy/MDMMDMA use in the last 30 days (0)**	2 (0.9)	1 (1.4)	6 (4.9)	2 (3.8)	0 (0)	χ^2^_1_ = 7.285	0.122
**Amphetamines/methamphetamine use ever in life (0)**	2 (0.9)	5 (7.1)	11 (9)	2 (3.8)	0 (0)	χ^2^_1_ = 16.411	0.003
**Amphetamines/methamphetamine use in the last 30 days (0)**	1 (0.4)	2 (2.9)	2 (1.6)	2 (3.8)	0 (0)	χ^2^_1_ = 5.095	0.278

**Table 6:** Association between the characteristics of the participants and Drug Use Module (consumption ever in life).

Variables		Drug Consumption ^a^ (*n* = 496)		Cannabis/Marijuana ^a^ (*n* = 496)		Cocaine ^a^ (*n* = 496)		Ecstasy/MDMMDMA ^a^ (*n* = 496)		Amphetamines ^a^ (*n* = 496)	
		OR ^b^ (95% CI)	*p* Value	OR (95% CI)	*p* Value	OR (95% CI)	*p* Value	OR (95% CI)	*p* Value	OR (95% CI)	*p* Value
**Nationality (Spain)**		N/A ^c^	<0.001	N/A	<0.001	N/A	0.072	N/A	0.002	N/A	N/A
	Belgium	1.066 (0.291–3.909)	0.923	1.120 (0.306–4.105)	0.864	0.164 (0.007–3.592)	0.251	0557 (0.057–5.448)	0.615	N/A	N/A
	France	2.625 (0.674–10.218)	0.164	2.458 (0.631–9.577)	0.195	3.034 (0.257–35.875)	0.379	0.960 (0.088–10.440)	0.974	N/A	N/A
	Brazil	5.092 (1.358–19.095)	0.016	4.895 (1.306–18.350)	0.018	3.282 (0.297–36.265)	0.332	3.343 (0.370–30.219)	0.283	N/A	N/A
	Others	0.519 (0.115–2.333)	0.392	0.431 (0.093–1.997)	0.282	1.625 (0.096–27.563)	0.737	0.595 (0.044–7.996)	0.695	N/A	N/A
**Age**		N/A	N/A	N/A	N/A	1.732 (1.116–2.689)	0.014	N/A	N/A	2.106 (1.412–3.142)	<0.001
**Gender**		N/A	0.001	N/A	0.001	N/A	0.012	N/A	N/A	N/A	N/A
	Male	3.094 (0.257–37.274)	0.374	2.996 (0.249–35.998)	0.387	0.159 (0.008–3.058)	0.223	N/A	N/A	N/A	N/A
	Female	1.167 (0.100–13.582)	0.902	1.150 (0.099–13.356)	0.911	0.051 (0.003-0.905)	0.043	N/A	N/A	N/A	N/A
**Problems as a result of drugs**		N/A	<0.001	N/A	<0.001	N/A	<0.001	N/A	<0.001	N/A	N/A
	Never	14.734 (1.835–118.307)	0.012	14.085 (1.756–112.974)	0.013	1.924 (0.219–16.883)	0.555	1.215 (0.147–10.076)	0.857	N/A	N/A
	Ever	206.980 (17.942–2387.690)	<0.001	208.633 (17.965–2422.935)	<0.001	20.463 (1.907–219.619)	0.013	15.598 (1.635–148.755)	0.017	N/A	N/A
		R^2 d^ = 0.255		R^2^ = 0.251		R^2^ = 0.353		R^2^ = 0.215		R^2^ = 0.126	

^a^ Logistic regression; ^b^ OR: odds ratio; ^c^ N/A: not applicable; ^d^ Nagelkerke’s R2.
